# Supplementary material for: Explain Breathlessness: Could ‘Usual’ Explanations Contribute to Maladaptive Beliefs of People Living with Breathlessness?
Source: Healthcare (Basel). 2024 Sep 10;12(18):1813. doi: 10.3390/healthcare12181813 (PMC11431128; doi:10.3390/healthcare12181813)
Supplement: Supplementary file 1 [file healthcare-12-01813-s001.zip › healthcare-3084084-supplementary.pdf]

Supplementary Materials

All open text responses to questions were coded using the framework developed and reported in Williams MT, Lewthwaite H, Brookes D, Jensen D, Abdallah S, Johnston KN. Breathlessness explanations and research priorities: Findings from an international Delphi survey. Journal of Pain and Symptom Management. 2020;59(2):310-319. [Note- Code categories not reported where n=0]

Table S1: Summary of descriptive content analysis of "usual explanation" for chronic breathlessness [Frequency of individual code within domains).

Domain	N (%) of respondents	Codes	Frequency n (% of responses
Breathlessness related to medical condition	40 (70%)	Due to your underlying pathology / medical condition	10 (18%)
		Because your lungs/heart are not working as well as they should (breathlessness caused by your illness/underlying condition)	31 [54%)
Physiological mechanisms of breathlessness	25 (44%)	To make sure your body/blood gets enough oxygen to fuel your muscles/keep up with body's needs	9 (16%)
		Increased respiratory rate =increased breathlessness	6 (11%)
		Trapped air in your lungs	4 (7%)
		Oxygen /Carbon dioxide levels	4 (7%)
		As people become weaker, increased breathlessness	4 (7%)
		Secretions in your lungs	3 (5%)
		You feel like not enough air is getting in or out; difficulty pumping air in or out/breathing pattern	3 (5%)
		Air tubes are tired and not as elastic	2 (4%)
		How good your muscles are at using oxygen	1 (2%)
Breathlessness is caused by lots of factors	17 [30%)	Same as domain name	
Possible contributing factors other than pathophysiological disease [Mechanisms, aggravating and relieving factors]	14 (25%)	sedentary /unfit /deconditioning	9 (16%)
		strenuous exercise	3 (5%)
		medications	3 (5%)
		extreme temperatures	1 (2%)
		Obesity	1 (2%)
		High altitude	1 (2%)
		Anemia	1 (2%)
		Past experiences	1 (2%)
		Thoughts/feelings	1 (2%)
		Perception generated by the brain from lots of inputs	1 (2%)
Degree of cure/control and strategies (Management principles)	12 (21%)	Strategies to manage/cope/live with	10 (18%)
		If we can't fix the problem, we can relieve the symptoms (optimized treatment but may still be breathless)	6 (11%)
		Some causes we can fix and some we can't	1 (2%)
Breathlessness is associated with panic/anxiety; makes it worse	8 (14%)	Same as domain name	
'Normalizing' breathlessness	7 (12%)	When we exert ourselves may become aware of breathing harder, deeper, faster than usual (Breathlessness is normal)	4 (7%)
		In some people sensation occurs early in physical exertion or without physical exertion, it is uncomfortable /concerning	2 (4%)
		Breathlessness is different for everyone	2 (4%)
		Normally breathing is not distressing and not noticed	1 (2%)
Breathlessness as a prompt to check if disease optimized	5 (9%)	Sign of a problem needing investigation	2 (4%)
		If symptoms worse than usual, check with health care provider er another reason	2 (4%)
		infection may cause breathlessness	1 (2%)
		Enquire whether they are following optimal medical advice	1 (2%)
Depends on pathology /Cannot explain until I knew the patient's condition*	4 (7%)	Same as domain name	
Breathlessness as sensation inconsistent relationship with internal state of lungs or test/scan results/oxygen levels	3 (5%)	Same as domain name	
Timeline of breathlessness	2 (4%)	Won't go away on its own /may get worse over time	1 (2%)
		Breathless over a longer period [chronic]	1 (2%)
Breathlessness does not mean you are in danger [Personal, not harmful but persists despite optimal treatment]	2 (4%)	Same as domain name	

*Percentage calculations out of n=57

Table S2: Summary of verbatim responses including differential phrases for changes to "usual explanation" for chronic breathlessness

Diagnosis /clinical situation	Examples of additional information to be included in explanations pending diagnosis/specific condition [verbatim]
Emphysema	• "Might talk about pipes - whether they are floppy and collapse" [ID 1]
COPD	• …during expiration, a loss of elasticity in the lungs, means the lungs do not empty air effectively, causing a sensation of effort with breathing. [ID 5]<br>• "Explain the disease e.g., for COPD would explain what it means to have an obstructive lung disease, that they have difficulty getting the air out of their lungs and that makes it hard for them to get another breath in" [ID 46]<br>• "COPD: Your breathlessness is most likely due to the trapped air in your lungs. Damage in your lungs makes it difficult for air to be pushed out during normal breathing and so a lot of it is left in your lungs. So when you go to take more breaths without attempting to fully empty your last breath you may not feel like you can get a good breath back in. We call this hyperinflation (and i show them the breathing result using accessory muscles). When this is the case the most important technique you can use is pursed lip breathing (and i demonstrate). The pressure of breathing out with this force is what is needed to let the trapped air escape so more can come in and you feel a relief of breath." [ID 56]<br>• "I explain in very simple terms what has happened to their lungs and why it makes the breathless. Always start by telling them where their lungs are and how they take up most of the chest. Most people don't' know this and are surprised. Eg copd, compare their lungs to broccoli, and how they are big sponges. Undamaged sponges when squashed bounce back fast, but sponges with big hole in them when squashed are slower to bounce back, so the tubes are squashed, and the air can't get out. Then talk about breathing and how the diaphragm works to create a negative pressure like a coffee vacuum pack. But if the lungs can't get the air out it builds up and if is more difficult to get the negative pressure." [ID 86]
Asthma	• "..irritated and annoyed, small and tight, plugged up with gunk" [ID 1]
IPF	• "With IPL, the air sacs in the lungs are thickened and stiff, and more effort is required to inflate the lungs. The increased effort required is felt as breathlessness. [ID 5]<br>• "If they have IPF their SOB is because their lungs are quite stiff and may need to take smaller breath" [ID 30]<br>• "Pulm fibrosis - different mechanism so I would be more explaining that the lungs are restricted and so they can't expand like they used to, and so the person can't get enough air in..." [ID 50]<br>• "Restrictive lung disease: the lungs do not expand to fully move air in and out (and i show them with my hand trying to breathe against a wall." [ID 56]<br>• Idiopathic fibrosis breathlessness can occur when the lungs become stiff, and sputum is hard to clear" [ID 58]<br>• "..with pulmonary fibrosis your lungs are scared and have no stretch to push air out, so when you move you will not always feel the low oxygen levels but you will feel breathless, to control this again borg scale used 3-4 and take lots of rest, where oxygen if prescribed." [ID 63]
Heart Failure	• "With heart failure, I explain that a reduction in the heart's pumping strength can cause fluid to accumulate in the walls lining the air sacs in the lungs, causing them to thicken and impair the transfer of oxygen that enters the lungs across the walls and into the blood stream. This can cause breathlessness." [ID 5]<br>• Features of the dyspnoea (e.g. orthopnea with heart failure), how it might change over time" [ID 25],<br>• "If they have heart failure, their SOB is related to pulmonary congestion rather than air trapping" [ID 30]<br>• "Add in the words heart is not pumping your blood to your muscles efficiently, so your muscles are not getting the energy they need. So your lungs need to breathe faster to get more oxygen to your body." [ID 31]<br>• "your heart is not working very well right now and even if it is trying to pum blood to your lungs or to the rest of the body, it could not remove all the fluid in your legs, this can sometime cause your legs to be puffy or swell or sometimes you are short of breath even if you are just sitting." [ID 32]<br>• "If heart failure is present, fluid may contribute, or chest infections may exacerbate breathless" [ID 33]<br>• "Heart failure: breathlessness can be caused from the weakness of your heart and lung vessels not pumping blood and oxygen as effective as needed. For example, you might have some floppy heart muscle which is damaged. Fluid can build up around your heart making it even more difficult to breathe." [ID 56]<br>• "Heart failure the breathlessness maybe due to the heart not beating effectively. Do you have any other symptoms of breathlessness at rest, lethargy, oedema, pounding heart." [ID 58]<br>• "Heart failure shortness of breath could be due to your heart being under pressure caused by fluid causing congestion in your lungs, this can reduce your oxygen exchange causing SOB. when you walk you may feel breathless as the heart is working a little harder also." [ID 63]
Neuromuscular disease	• Features of the dyspnea, how it might change over time, what this might mean (if they have aggressive cancer, it may be less important, if they have MND it is likely to be the most important marker of prognosis" {ID 25])<br>• "Neuromuscular disease: breathlessness is caused from the incomplete or absent messages of the brain to respond to the activity you do. Generally, the lack of muscle tone reduces the amount of air that can be used as well as you would like." [ID 56]
Cystic fibrosis	• "Cystic Fibrosis: breathlessness is often caused by the damaged breathing tubes (assuming this is bronchiectasis CF). The breathing tubes become floppy in places and loose the elasticity to push out sputum (sort of like 10-year-old pair of undies compared to a new pair). Sputum gets trapped and this reduces oxygen flow as well as like." [ID 56]

Table S3: Summary of descriptive content analysis of "important to include "in a usual explanation" for chronic breathlessness [Frequency of individual code within domains).

Domain	Frequency n (% of responses)	Codes	Frequency n (% of responses
Acknowledge and validate	12 (21%)	the distress / suffering caused by this symptom and impact on emotions	8 (14%)
		limitations, disability and impact on daily life	4 [7%)
		that the sensation is always unpleasant or uncomfortable	2 (4%)
		that it is a legitimate concern to bring to clinical attention	1 (2%)
Person centered communication:	3 (5%)	Include everything, but delivered in an appropriate time frame depending on patients wish for information, ability to understand and general condition	2 (4%)
		Use both a medical term and a plain language term	1 (2%)
		Emphasize to the patient the importance of expressing his/her fears and concerns (NEW item)	1 (2%)
Personal, not harmful but persists despite optimal treatment:	21 (37%)	that it is not harmful in itself but should be reported if breathlessness becomes more frequent or severe [seek medical advice is unwell]	5 (9%)
		that all reversible (treatable) causes of breathlessness have been sought	2 (4%)
		that being breathless from exertion isn't harmful [does not indicate danger/harm]	2 (4%)
		that breathlessness is a personal, lived experience of a sensation	1 (2%)
		that breathlessness is part of most people's normal life, is an expected reaction to a situation but chronic breathlessness is out of proportion to activity, provoked by lesser activities/stress and feels worse. Normalize	12 (21%)
		that in most instances does not closely reflect hypoxemia or suffocation [supp oxygen will not help in many cases]	6 (11%)
Mechanisms, aggravating and relieving factors:	21 (37%)	that it can be exacerbated by unhelpful emotions/thoughts such as anxiety/worry	5 (9%)
		that there are different physiological origins	4 (7%)
		that there are related factors can precipitate or alleviate the sensation	3 (5%)
		that it is multi-factorial, not just due to the underlying condition	3 (5%)
		that the brain is central to sensation	1 (2%)
		the lung pathology and the exact vicious circles of emotions and behaviors that are relevant to that individual patient [use for responses which indicate cause is their disease/condition]	10 (18%)
		the vicious circle of decreased activity, compounded by muscle inactivity and deconditioning and further worsening breathlessness	1 (2%)
Symptomology (variability and trajectory):	2 (4%)	there will be a continual decline in functional status and what that may look like	2 (4%)
Management principles:	36 (63%)	it is something that people can live with and self-manage	2 (4%)
		the importance of staying active	5 (9%)
		it is not possible to eliminate it completely but that it may be possible to eliminate some of the things contributing to it and it is possible to change reaction to it, cope, adapt and self-manage [coping techniques, breathing control, pacing]	7 (12%)
		there are treatment options to manage dyspnea (to relieve the distress/ intensity/ impact) and improve quality of life	
		support groups are available so know they are not alone	1 (2%)
		we can't take the breathlessness away, but we can look to impact how we think, feel and behave.	2 (4%)
		strategies may include non-pharmacological approaches including psychotherapeutic like approaches	1 (2%)
		there may be barriers towards opioid treatment	1 (2%)

Percentage calculations out of n=57

Table S4: Summary of descriptive content analysis of "important to avoid" in a usual explanation" for chronic breathlessness [Frequency of individual code within domains).

Domain	Frequency n (% of responses)	Codes	Frequency n (% of responses
Chronic Breathlessness is not	21 (60%)	That breathlessness is 'in your head", only in the mind and therefore imaginary/not as "hard" as other measurements such as lung function [you shouldn't/are not breathless]	6 (17%)
		that this is a sign of impending death	4 (11%)
		we don't know why people get breathless as the correlation to other clinical tests (breathing frequency, heart rate, saturation, lung function) is low	1 (3%)
		that this means that the oxygen saturation in the blood is definitely low or can be relieved by oxygen as a first-choice treatment option [Oxygen will relieve]	12 (34%)
Specific terms	6 (17%)	"panic" or "panic disorder"	1 (3%)
		"intractable" /untreatable	1 (3%)
		"Oxygen dependent"	1 (3%)
		"Choking, drowning, suffocating"	1 (3%)
		"Dyspnea, respiratory failure, respiratory depression"	1 (3%)
		"Oxygen, carbon dioxide"	1 (3%)
		"heart/lung injured /damaged"	2 (6%)
		the idea of taking breathlessness away completely	2 (6%)
		saying that all breathlessness should be avoided	5 (14%)
		focusing on deep/big breaths when recovering	1 (3%)
Blaming and hopelessness	8 (23%)	saying anything that implies that there is nothing (more) that can be done for it	6 (17%)
		blaming the person or letting them blame themselves and at the same time encouraging them to actively take part in their own self-management with support from others (NEW item)	2 (6%)

Responses calculated out of respondents indicating yes (n=35)

Table S5: Summary of responses for concepts which people living with chronic breathlessness find difficult to understand.

Domain	Codes	Frequency n (% of responses
>11 respondents	Inconsistent relationship between Sa/PaO_2/Supp O_2/CO_2 and breathlessness	19 (39%)
6 to 10 respondents	Relationship between exercise training /inactivity and breathlessness	8 (16%)
	Pathophysiology/anatomy of chronic condition	6 (12%)
	Chronic condition cannot be improved/ breathlessness cannot be cured	6 (12%)
5 respondents or less (≤10%)	Specific reason /cause of their breathlessness //not understanding multifactorial	5 (10%)
	complicated explanations and medical terminology	5 (10%)
	That their experience of breathlessness can be improved /aim is to manage symptoms)	5 (10%)
	breathlessness not harmful in itself /Increase breathlessness = increased damage/harm	4 (8%)
	role of anxiety and breathlessness	3 (6%)
	relationship between fluid overload and breathlessness	3 (6%)
	still breathlessness despite optimal management	3 (6%)
	how non pharmacological treatments help/work	2 (4%)
	medications	2 (4%)
	relationship between smoking history and breathlessness	2 (4%)
	Mechanics of breathing	2 (4%)
	Can be functional despite breathlessness	2 (4%)
	Trajectory of breathlessness (rapid deterioration)	1 (2%)
	role of opioids	1 (2%)
	Ascribe alternate primary reason for breathlessness (e.g. unfit, age)	1 (2%)

Responses calculated out of respondents indicating yes (n=49)
